# Low genetic differentiation among morphologically distinct *Cycas* species informs the delineation of conservation management units

**DOI:** 10.1093/aob/mcaf276

**Published:** 2025-11-13

**Authors:** James A R Clugston, Nicholas J Cuff, Caroline Chong, Michael Calonje, Kayla Claravall, Rachael V Gallagher, Murray Henwood, Gregory J Kenicer, Richard Milne, Markus Ruhsam

**Affiliations:** Hawkesbury Institute for the Environment, Western Sydney University, Penrith, NSW 2751, Australia; Montgomery Botanical Center, Coral Gables, FL 33156-4242, USA; Flora & Fauna Division, Northern Territory Department of Lands, Planning and Environment, Palmerston, NT 0830, Australia; Flora & Fauna Division, Northern Territory Department of Lands, Planning and Environment, Palmerston, NT 0830, Australia; Montgomery Botanical Center, Coral Gables, FL 33156-4242, USA; The University of Sydney, School of Life and Environmental Sciences, Camperdown, NSW 2006, Australia; Hawkesbury Institute for the Environment, Western Sydney University, Penrith, NSW 2751, Australia; The University of Sydney, School of Life and Environmental Sciences, Camperdown, NSW 2006, Australia; Royal Botanic Garden Edinburgh, Edinburgh EH3 5LR, UK; The University of Edinburgh, College of Science and Engineering, School of Biological Sciences, Institute of Molecular Plant Sciences, Edinburgh EH9 3JN, UK; Royal Botanic Garden Edinburgh, Edinburgh EH3 5LR, UK

**Keywords:** Cycads, *Cycas*, genomics, conservation genomics, Australia, management units

## Abstract

**Background and Aims:**

Cycads are the most threatened group of seed plants, with isolation and habitat fragmentation among the primary drivers of species decline. Understanding how genetic diversity is distributed across populations is crucial for informing conservation management and identifying genetically vulnerable populations that require conservation attention.

**Methods:**

Here we investigated the genetic diversity and structure of two endemic Australian species of significant conservation concern, *Cycas armstrongii* and *C. maconochiei* subsp. *maconochiei*. Two hundred and thirty-six individuals were sampled from 26 populations across their native ranges, including a presumed putative hybrid population (*C. armstrongii × maconochiei*), utilizing next-generation sequencing in the form of restriction site-associated DNA sequencing (RADseq).

**Key Results:**

Our results suggested low levels of genetic diversity in both taxa (*C. armstrongii*, *H*_e_ ≤ 0.038; *C. maconochiei* subsp. *maconochiei*, *H*_e_ ≤ 0.061) and no evidence for inbreeding (mean *G*_IS_ −0.143 and −0.153, respectively). Analysis of molecular variance indicated minimal genetic differentiation between populations (2.41 %) and between taxa (1.81 %). However, pairwise *F*_ST_ values and the Mantel test revealed significant isolation by distance (*r* = 0.606, *P* < 0.0001). Discriminant analysis of principal components and popuatlion STRUCTURE analysis indicated admixture, between populations. Morphological traits, principal component and environmental analysis based on seven traits found significant differentiation in five characters, four of which were environmentally linked. The results showed no clear signal of interspecific hybridization for either taxon.

**Conclusions:**

These findings indicate *C. armstrongii* and *C. maconochiei* subsp. *maconochiei* likely represent a morphologically variable species. In addition to updating the threat assessment, we recommend: (1) formally recognizing genetically depauperate or geographically isolated populations (e.g. Tiwi Islands) as conservation management units (CMUs); (2) establishing new *ex situ* assurance collections for at-risk CMUs; and (3) implementing assisted gene flow among genetically compatible populations to enhance adaptive potential. These actions will ensure conservation strategies are tailored to evolutionary and ecological units.

## INTRODUCTION

Cycads (Cycadales) are among the oldest living seed plants and are of global conservation concern (Donaldson, 2003). While noted for their evolutionary significance, cycads are also considered one of the most threatened plant groups worldwide, with ∼70 % of extant species currently at risk of extinction (IUCN, 2025). Cycad vulnerability to extinction is further compounded by their extremely slow rates of growth and reproduction, with some species taking decades to reach maturity (Raimondo and Donaldson, 2003). In addition, anthropogenic pressures such as over-collection for horticulture, habitat loss due to land use change, increasing climate extremes and population fragmentation have all contributed to dramatic declines in cycad populations worldwide (Donaldson, 2003). The compounding impacts of these threats create a significant risk to the persistence of cycad populations and species in the wild without new efforts to undertake targeted conservation interventions (Mankga and Yessoufou, 2017).

Effective conservation strategies for cycads and other threatened plant taxa must incorporate genomic data to understand population-level genetic diversity, distinctiveness and differentiation, which are critical for defining conservation management units (CMUs), that prioritize populations based on genetic uniqueness and ecological importance (Palsbøll *et al.*, 2007; Coates *et al.*, 2018). This approach is critical in cycads, where limited seed dispersal and slow reproductive rates restrict gene flow, increase the effects of genetic drift, and often lead to low within-population genetic diversity along with high differentiation among populations (González-Astorga *et al*., 2008; Zhan *et al*., 2011; Swart *et al.*, 2018; Tobgay *et al.*, 2023). In addition, species delimitation in cycads is complicated by widespread morphological plasticity and frequent hybridization (Keppel, 2009; Chiang *et al.*, 2013; Tao *et al.*, 2021; Liu *et al.*, 2022*a*, *b*). Many diagnostic traits are environmentally variable and not consistently identifiable in the field (Newell, 1985; Calonje *et al.*, 2014), which makes morphology alone an unreliable basis for species classification (Hill, 1998). This issue also extends beyond cycads, with notable examples including *Eucalyptus* (e.g. McKinnon *et al.*, 2008), where extensive hybridization and phenotypic plasticity blur species boundaries, and *Quercus* (e.g. Kremer *et al.*, 2002), where interspecific gene flow and convergent leaf morphology complicate taxonomic resolution. Similarly, in *Salix*, morphological traits can vary with environmental conditions, and frequent hybridization makes species-level identification difficult without genetic data (Hardig *et al.*, 2000). In areas of sympatry, closely related taxa often hybridize due to overlapping ranges and shared pollinators, producing morphologically intermediate individuals and blurring taxonomic boundaries (Chiang *et al.*, 2013; Alix *et al*., 2017). Although hybridization can introduce novel genetic variation, it may also erode species integrity, complicating conservation efforts and management (Ellstrand *et al.*, 1989). These challenges highlight the limitations of morphology-based taxonomy and underscore the importance of genomic data in informing accurate species delimitation and guiding targeted, evidence-based conservation planning in cycads.

Australia is recognized globally as a biodiversity hotspot, with approximately between 88 and 92 % of its vascular plant species being endemic to the continent (Gallagher *et al.*, 2023). This exceptional level of endemism extends to its cycad flora. The country exhibits the highest species richness of cycads globally, with four genera present: *Macrozamia*, *Lepidozamia*, *Bowenia* (all endemic) and *Cycas*. Notably, Australia contains the greatest species diversity in the genus *Cycas*, with 34 native species, of which 31 are endemic. Despite this remarkable richness, only a handful of species in the genus, such as *C. megacarpa* and *C. calcicola*, have been studied in detail at the population genetic level (Clugston *et al.*, 2016; James *et al.*, 2018). However, cycads in Australia face increasing threats from habitat conversion, fragmentation, invasive species, seed overharvesting and climate change (Sax *et al.*, 2002; Liddle, 2009; Spooner *et al.*, 2018). These pressures impact species richness and community composition, disrupting ecological processes and evolutionary trajectories of species, populations and ecological communities (Shaw *et al.*, 2025).

A reduction in intraspecific and interspecific gene flow within and between cycad populations can lead to low levels of genetic variation and increase the likelihood of population decline (Ellstrand, 1993). This issue is particularly concerning given the increasing threats on populations, which can significantly impact species with slow life histories (Marler and Ferreras, 2017). For example, high levels of genetic differentiation across populations, along with low diversity within individual populations, has been reported for *Cycas balansae* and *C. seemannii* (Keppel *et al.*, 2002; Long-Qian and Xun, 2006). Clugston *et al.* (2022) noted considerable genetic variation and isolation in the Australian cycad *C. calcicola* across major geographic regions, with many populations exhibiting some degree of inbreeding. Similar patterns have been observed in other plant groups, both within Australia and internationally. For example, in Australia, *Banksia* species exhibit strong genetic structuring and an increase in inbreeding due to habitat fragmentation (He *et al.*, 2004). In Europe, *Primula vulgaris* shows reduced genetic diversity in isolated populations due to limited pollen and seed dispersal (Jacquemyn *et al.*, 2009), while *Helianthemum nummularium* displays significant genetic drift driven by isolation by distance and small population size (Beatty *et al.*, 2008). These examples highlight how geographic isolation and restricted gene flow can shape genetic structure across different taxa and regions.

To improve conservation outcomes for cycads, it is essential to carefully delineate and confirm species boundaries, understand genetic diversity, and define conservation management units based on robust species concepts (Rossetto *et al.*, 2021). Achieving this requires integrating morphological, ecological and molecular data to capture the full spectrum of interspecific and intraspecific variation (Duminil and Di Michele, 2009). In cycads, inconsistencies between morphological traits and genetic markers often complicate taxonomic classification, suggesting that current species circumscriptions may be inadequate (Chang *et al.*, 2022). Cycads have been shown to have undergone a recent evolutionary radiation (Nagalingum *et al.*, 2011), and more recent fossil-integrative analyses confirm that, despite a crown origin in the late Carboniferous (∼330 million years ago), many extant species diverged within the last 5–15 million years (Coiro *et al.*, 2023).

Traditional molecular tools such as DNA barcoding, which use limited genetic markers, are often insufficient to resolve species boundaries in cycads due to their low levels of sequence divergence (Sass *et al.*, 2007; Hollingsworth *et al.*, 2011; Nicolalde-Morejón *et al.*, 2011). However, next-generation sequencing approaches such as restriction-site associated DNA sequencing (RADseq) allow the generation of thousands of genome-wide markers and can uncover fine-scale genetic structure, even in recently diverged taxa (Eaton and Ree, 2013; Gramlich *et al.*, 2018). RADseq has proven effective in resolving phylogenetic relationships, detecting hybridization and identifying backcrosses in plant lineages, including those with large and complex genomes, such as cycads (Clugston *et al.*, 2016).

Here, we investigate the genetic diversity, differentiation and potential hybridization between two cycad taxa from northern Australia: *Cycas armstrongii* and *Cycas maconochiei* subsp. *maconochiei*, using genome-wide SNP data from RADseq and complementary morphological analyses of leaf and cuticle traits. Our study addresses an urgent conservation need in the Greater Darwin region by clarifying species boundaries and evaluating the degree of genetic and morphological distinctiveness between these sympatric and morphologically similar taxa. Specifically, we aim to identify CMUs, assess the extent of hybridization, and provide a framework to inform species delimitation, conservation status assessments and future management actions. In doing so, we contribute to a broader understanding of species boundaries, evolutionary processes and conservation priorities in one of the world’s oldest and most threatened plant lineages.

## MATERIALS AND METHODS

### Study species

**Fig. 1.**
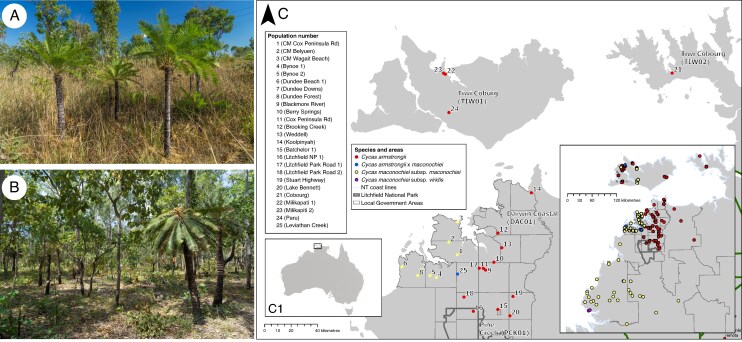
Images and distribution of herbarium collections (inset map) and samples collected as part of the current study for *Cycas armstrongii* and *C. maconochiei* subsp. *maconochiei* (main map). (A) Populations of *C. armstrongii* growing along the Stuart Highway, in the Darwin Region, Northern Territory, Australia. (B) Population of *C. maconochiei* subsp. *maconochiei* growing in the Darwin Cox Peninsula. (C) Map illustrating the northern section of Australia’s Northern Territory. Inset map (C1) shows the map footprint in relation to Australia. The primary map displays the population-level sample distribution from key areas, including Darwin, Cox Peninsula, Dundee Beach, Cobourg Peninsula and the Tiwi Islands. These locations fall within the Interim Biogeographic Regionalisation for Australia (IBRA) subregions – Darwin Coastal (DAC01), Pine Creek (PCK01), Cobourg (TIW01) and Tiwi (TIW02) – showcasing the majority of populations and a hybrid population of C*. armstrongii × maconochiei* in the Leviathan Creek area. The inset map presents the complete distribution of all taxa in the species complex, highlighting the lack of population-level data for the southern range of *C. maconochiei* subsp. *maconochiei* and *C. maconochiei* subsp. *viridis*.

*Cycas armstrongii* and *Cycas maconochiei* subsp. *maconochiei* are among the most widespread cycads across the ‘Top End’ (Fig. 1A–C) in the Northern Territory (NT) of Australia. Both species are locally abundant yet occur in scattered populations, often in *Eucalyptus tetrodonta* open forests on lateritic kandosols. While *C. armstrongii* is listed as Vulnerable under Northern Territory legislation, neither species is currently listed as threatened under national or IUCN frameworks. *Cycas armstrongii* ranges from the Wagait Beach through the Greater Darwin region to the Mary River, with disjunct populations on the Tiwi Islands and Cobourg Peninsula. It often forms dense stands, particularly where it dominates the midstorey in tall open forests, whereas *C. maconochiei* subsp. *maconochiei* is primarily found along the north-west coast of the Top End, including the Fitzmaurice and Daly River catchments, Cox Peninsula and the Tiwi Islands. It is distinguished from *C. armstrongii* by darker green, recurved leaflets and the lack of a terminal point on cataphylls (*sensu* Hill, 1996).

The two taxa co-occur in several regions and are part of a broader species complex known to hybridize (Hill, 1996). Both are insect-pollinated and have limited seed dispersal (<5 m), likely restricting gene flow and contributing to population-level genetic differentiation (Watkinson and Powell, 1997; Clugston *et al.*, 2016). It is important to note that two subspecies are recognized within *C. maconochiei sensu lato*: *C. maconochiei* subsp. *maconochiei* and *C. maconochiei* subsp. *viridis*, the latter of which is restricted to the Fossil Head/Kumbunbar Creek area south of Wadeye (Dixon, 2004; Australian Plant Name Index, 2025).

### Sampling strategy

Fresh leaflets were placed in silica gel, and herbarium specimens were collected from wild populations of *C. armstrongii*, *C. maconochiei* subsp. *maconochiei* and a perceived putative hybrid population (*C. armstrongii × maconochiei sensu* Hill, 1996), from the Northern Territory, Australia (Fig. 1C). Populations were selected based on previously published records by Liddle (2009), Dixon (2004) and Hill (1994) and herbarium specimens held by The New South Wales National Herbarium (NSW), Northern Territory Herbarium (DNA), and observations from the Australasian Virtual Herbarium (AVH) (https://avh.chah.org.au, accessed 30 March 2017). It should be noted that these selected populations approximate the known geographic extent of *C. armstrongii*, but only the northern part of the known geographic extent of *C. maconochiei* subsp. *maconochiei* (Hill, 1996; Fig. 1C), and populations collected in the Tiwi Cobourg region were identified as *C. armstrongii*. All significant stands of what Hill (1996) originally circumscribed as *C. maconochiei* subsp. *lanata* (currently recognized as synonym of *C. maconochiei* subsp. *maconochiei*) occur further south in the Darwin Coastal bioregion from the Daly River mouth to the Port Keats area and into the adjacent Victoria Bonaparte bioregion, with a geographic gap in the distribution of the subspecies as now circumscribed of ∼70 km between Dundee Beach and Daly River mouth. This study does not include samples from the southern parts of the taxon’s distribution.

Collections totalled 226 individuals from 25 populations (approximately ten samples per population; Supplementary Data Table S1, Fig. 1C) for *C. armstrongii* (*n* = 142) and *C. maconochiei* subsp. *maconochiei* (*n* = 74), and a population of suspected hybrids (= *C. armstrongii × maconochiei* (*n* = 10)). The sampled populations represent the following biogeographic regions and subregions (in parentheses) in Northern Territory modified from the Interim Biogeographic Regionalisation for Australia (IBRA7; Australian Government, Department of the Environment and Heritage, 2000) by experts from the Northern Territory Department of Lands, Planning and Environment (B. Lynch, Northern Territory Department of Lands, Planning and Environment, Australia, pers. comm.): Darwin Coastal (DAC01), Pine Creek (PCK01) and Tiwi Cobourg (TIW01 and TIW02). Populations of *C. maconochiei* from the Daly Basin (IBRA subregion DOB01) were not sampled due to access constraints.

### Molecular methods

#### DNA extraction and quantification

Approximately 0.05 g of silica-dried leaflets were ground using a TissueLyser (Qiagen, Hilden, Germany). High molecular weight genomic DNA was extracted using a DNeasy Plant DNA Mini Kit (Qiagen, Hilden, Germany). Genomic DNA was inspected using a 2 % agarose gel to check for the presence of DNA and impurities, and then DNA extractions were quantified using an Invitrogen Qubit (3.0 BR DNA assay; Invitrogen, Life Technologies, Carlsbad, CA, USA) fluorometer with a target concentration of 17 µg mL^−1^. Any samples that yielded less than this amount were re-extracted or concentrated using a 1:1 ratio of Agencourt AMPure XP sample purification beads (Beckman Coulter, Inc.) by combining multiple extractions from the same sample.

#### DNA normalization and restriction digest reaction

Genomic DNA was normalized to a concentration of 500 ng in 42 µL total volume (0.01 µg mL^−1^), then 5 µL of NEB 10× CutSmart buffer (New England Biolabs, Ipswich, MA, USA) and 1 µL of bovine serum albumin (BSA) were added to each well. Samples were held at 4 °C for a minimum of 5 h before adding restriction enzymes to improve the cutting action of the restriction enzymes. Double digest reactions were carried out using 1 µL each of the restriction enzymes EcoR1-HF and Mse1. Reactions were placed into a thermocycler for 3 h at 37 °C with a final 20-min enzyme deactivation step at 65 °C. Digests were checked on a 2 % agarose gel for quality of digestion. The reactions were cleaned using a 1.8:1 ratio of AMPure XP beads to sample (90 µL of AMPure XP beads to 50 µL of digested DNA) and quantified using a Qubit high-sensitivity kit.

#### Library preparation

Libraries were prepared using an Illumina TruSeq Nano High-Throughput Dual Index Library Preparation Kit (Illumina Inc., CA, USA). We followed the ezRAD v.3 method (Toonen *et al.*, 2013), using half of the recommended volumes of the kit to reduce costs. Following Clugston *et al.* (2016), the final steps of library preparation were modified from the ezRAD protocol by using a final bead clean with a 0.8:1 ratio of AMPure XP beads to remove adapter dimer. The final Illumina libraries were then validated using a LabChip and cleaned using a 0.9:1 ratio of AMPure XP beads. Samples were then quantified again using a Qubit high sensitivity kit. Final libraries were normalized to a concentration of 10 nm, after which 5 µL of library was pooled for sequencing.

#### Sequencing

Following Clugston *et al.* (2016), we aimed to capture ∼1 GB of sequence data per sample (in three runs of 96 libraries, including 142 samples of *C. armstrongii*, 74 samples of *C. maconochiei* subsp*. maconochiei* and 10 samples of *C. armstrongii × maconochiei*). Our goal was to obtain adequate coverage of the large genome for all species in this study and capture as much of the nuclear genome as possible while accounting for the over-representation of the plastid genome. Sequencing was completed using an Illumina HiSeq 4000, with 150 bp paired-end on two lanes and spiked with 10 % Phix sequencing control V3 (sequencing and quility control (QC) completed by Ramaciotti Centre for Genomics, NSW, Australia).

### Bioinformatics

#### Quality control and assembly of RADseq data

Illumina paired-end reads were assessed for quality using FastQC v.0.11.4 (Andrews *et al*., 2010). Subsequent read assembly and filtering were performed using ipyrad v.0.9.92 (Eaton and Overcast, 2020), with reads mapped to the reference genome of *Cycas panzhihuaensis* (Liu *et al.*, 2022*a*, *b*). Within ipyrad, the first and last five bases of both forward and reverse reads were trimmed, and bases with a Phred quality score <33 were converted to ‘N’. Reads containing ≥15 uncalled bases were discarded, and the data type was set to pairgbs. Adapter sequences were filtered and trimmed and reads shorter than 50 bp after trimming were removed.

Reads were clustered using a similarity threshold of 0.90 to account for close genetic relatedness among samples. Both the minimum depth for statistical base calling and majority-rule base calling were set to 6. The maximum proportion of uncalled bases per consensus sequence was set to 0.1 for both forward and reverse reads. The maximum shared heterozygosity per locus was left at the default value of 0.5 to minimize the impact of potential paralogues. Loci were retained only if they were present in at least 113 samples (≥50 % of individuals), ensuring sufficient coverage for downstream population-level analyses (Shafer and Thomson, 2007). All other parameters were left at ipyrad defaults. The resulting SNP dataset was further filtered using VCFtools v.0.1.16 (Danecek *et al.*, 2011) to exclude loci with >50 % missing data across samples.

#### Population genetic statistics and genetic distance

The following population genetic statistics were calculated using GenoDive v.3.06 (Meirmans, 2020): number of alleles observed in each population (*N*), effective number of alleles (Eff*N*), observed heterozygosity (*H_o_*), expected heterozygosity (*H*_e_), inbreeding index (*G*_IS_) and pairwise *F*_ST_ values using the Codom-Allelic option with 999 permutations. As estimates of heterozygosity based on SNP data can be biased by sample size and the preferential selection of polymorphic sites, we included both monomorphic and polymorphic loci in the analysis following Lachance and Tishkoff (2013). Levels of genetic differentiation among IBRA7 bioregions (Department of the Environment, Australian Government, 2012), between populations and species, were inferred using an analysis of molecular variance (AMOVA) in poppr v.2.9.6 (Kamvar *et al.*, 2014) in R v.4.4.1 (R Core Team, 2023). To evaluate the relationship between genetic differentiation and geographic distance, a Mantel test was performed using vegan 2.7-1 in R (Oksanen *et al.*, 2001). Input consisted of pairwise *F*_ST_ values generated in GenoDive and geographic distances (in kilometres) calculated from the centroid coordinates of each sampling locality using the Haversine method implemented in geosphere v.1.5–20 in R (Hijmans, 2010). The Mantel test was performed using Pearson’s product-moment correlation with 9999 permutations to assess statistical significance.

#### Population structure

STRUCTURE v.2.3.4 (Pritchard *et al.*, 2000) was used to explore the genetic structure and identify the most likely number of distinct genetic groups. The analyses were carried out for *K* = 1–25 using 1 000 000 Markov chain Monte Carlo (MCMC) iterations after a burn-in of 100 000 steps and were repeated 20 times for each *K*. To identify the most likely number of distinct genetic groups (*K*), Ln Pr[*X*|*K*] and Δ*K* statistics were calculated and visualized using the web-based StructureSelector (Li and Liu, 2018). The Δ*K* statistic (Evanno *et al.*, 2005) is based on the rate of change in the log probability of data between successive *K* values. A discriminant analysis of principal components (DAPC) was carried out in adegenet 2.1.10 (Jombart and Ahmed, 2011) to visualize the genetic relationships among populations. The optimal number of clusters in the data and the number of principal components/axes (PCAs) to be retained for discriminant analysis were determined using the find.clusters command in combination with the optimal a-score. A DAPC scatter plot was used to depict the relationships and connectivity of individuals.

### Morphological methods

#### Leaf cuticle sample preparation

For each sample, 10–15 (depending on leaf size) circular leaf discs with a diameter of ∼3 mm were removed from leaflets using a stainless-steel punch. Following the proposed methods by Alvin and Boulter (1974) for gymnosperm leaf cuticle analysis, 6 mL of a 20 % chromic acid (CrO^4^) solution was added to the glass vials containing the leaf disc samples and they were stored in a fume hood for 96 h at room temperature (around 22 °C). After the 96-h pretreatment process, the isolated leaf cuticles were removed from the solution, washed with distilled water and left to air-dry on filter paper for a minimum of 4 h. Dried leaf cuticle samples were then examined individually using a dissecting microscope, selecting a single adaxial and abaxial cuticle. Selected samples were mounted onto Agar Scientific 0.5″ aluminium specimen pin stubs (Agar Scientific Ltd, Essex, UK) using 12-mm Agar Scientific carbon adhesive tabs. The mounted stubs were then sputter-coated with 100 % platinum (Pt) for 3 min using an Emitech K575X sputter coater (PolyK Technologies, LLC, State College, USA). Scanning electron microscopy was carried out using a Zeiss Leo Supra 55 VP instrument (Carl Zeiss, Oberkochen, Germany) and the internal adaxial leaflet surface was imaged at a magnification of ×500. Additionally, leaf morphological measurements were taken from herbarium specimens for the following herbaria: Royal Botanic Garden Edinburgh (EDI), The New South Wales National Herbarium (NSW), Northern Territory Herbarium (DNA) and the National Herbarium of Victoria (MEL).

#### Morphological measurements

Measurements of both the leaf cuticle and leaves were carried out using ImageJ 1.52r (Rueden *et al.*, 2017), with only variable characters being selected for all species following the methodology proposed by Vovides *et al.* (2018). A total of seven characters were measured from the leaf and leaf cuticle. Measurements from the leaf were angle of insertion of the median leaflet to the rachis (angle of insertion to ML (°)), width of the median leaflet (ML width, mm) and length of the median leaflet (ML length, mm). Measurements from the leaf cuticle were area of the stomatal apparatus (Area Stom Ap (μm)), length of left guard cell (LLGCell (μm)), width of left guard cell (WLGCell (μm)) and length of upper polar extension (LUPolar (μm)).

#### Morphological analysis

Statistical analysis was completed using R 3.3.2 (R Core Team, 2019). Summary statistics were produced for three informative traits: area of stomatal apparatus, length of left guard cell and width of left guard cell. Pairwise *t*-tests were carried out to test each character for statistical significance between taxa (*C. armstrongii*, *C. maconochiei* subsp. *maconochiei* and *C. armstrongii × maconochiei*). Then traits were visualized using boxplots.in ggplot2 3.5.2 (Wickham, 2016). Each boxplot shows the median (horizontal line), interquartile range (boxes) and potential outliers (points). To identify informative characters and visualize character differentiation, a principal component analysis was carried out using the vegan community ecology package v.2.5-6 (Oksanen, 2001) and ggbiplot v.0.55 (Vu, 2024).

To assess the relationship between morphological variation and climatic conditions across sites, we extracted six bioclimatic variables (BIO1, BIO4, BIO5, BIO6, BIO12 and BIO15) from the WorldClim 2.1 dataset at 10 arc-min resolution (Fick and Hijmans, 2017). Site-level trait means were computed for seven traits: angle of insertion of the median leaflet, width and length of the median leaflet, stomatal apparatus area, length and width of the left guard cell, and length of the upper polar extension. We then computed pairwise Pearson correlation coefficients (*r*) and associated *P*-values using cor.test() in R. The resulting correlation matrix was visualized as a heat map using the ggcorrplot package (Kassambara, 2019), with significant associations (*P* < 0.05) denoted by asterisks.

## RESULTS

### Sequencing and *de novo* assembly

After sequencing raw reads ranged from 2 240 704 to 16 253 580 (mean = 3 913 537.33), and after the initial filtering of reads in ipyrad the number of reads that passed quality control filters ranged from 2 217 858 to 15 703 729 (mean = 3 852 333.123) per sample. *De novo* assembly of the reads generated sequence clusters from 1 040 592 to 2 722 158 (mean = 1 451 970.47) with 3180–354 886 (mean = 16 604.57) clusters containing six or more reads and being hi-depth. This resulted in a sequence read consensus ranging from 1293 to 110 548 (mean = 4517.33) with 2635 SNPs present in at least 50 % of all individuals (113 minimum samples per locus).

### Population genetic statistics and genetic distance

For *Cycas armstrongii*, observed heterozygosity (*H*_o_) ranged from *H*_o_ = 0.028 in Paru to *H*_o_ = 0.043 in Litchfield Park Road 1, with a mean of *H*_o_ = 0.035. Expected heterozygosity (*H*_e_) ranged from *H*_e_ = 0.025 in Paru to *H*_e_ = 0.034 in Weddell (Table 1), indicating low levels of genetic diversity in *C. armstrongii.* The inbreeding coefficient (*G*_IS_) ranged from *G*_IS_ = −0.107 in Litchfield Park Road 1 to *G*_IS_ = −0.199 in Lake Bennett, with an average across all populations of *G*_IS_ = −0.143. None of the *G*_IS_ values (mean = −0.143) significantly differed from zero (Table 1).

**Table 1.** Summary of population genetic statistics for *Cycas armstrongii* populations.

Population	*n*	Eff*N*	*H*_O_	*H_e_*	*G*_IS_
Milikapiti 1	1.131	1.035	0.032	0.029	−0.133
Milikapiti 2	1.158	1.041	0.038	0.033	−0.141
Paru	1.106	1.031	0.028	0.025	−0.125
Cobourg	1.102	1.040	0.036	0.031	−0.141
Koolpinyah	1.129	1.036	0.033	0.028	−0.148
Brooking Creek	1.148	1.042	0.038	0.033	−0.135
Weddell	1.147	1.045	0.039	0.034	−0.162
Cox Peninsula Road	1.141	1.041	0.036	0.031	−0.149
Berry Springs	1.102	1.037	0.033	0.029	−0.145
Blackmore River	1.117	1.042	0.037	0.032	−0.143
Litchfield Park Road 1	1.137	1.036	0.032	0.029	−0.107
Litchfield Park Road 2	1.119	1.035	0.032	0.028	−0.148
Lake Bennett	1.125	1.043	0.039	0.032	−0.199
Batchelor	1.131	1.042	0.037	0.033	−0.128
Litchfield NP	1.156	1.048	0.043	0.038	−0.119
Stuart Highway	1.106	1.035	0.032	0.027	−0.165
Total (mean)	1.128	1.039	0.035	0.030	−0.143

For *Cycas maconochiei* subsp*. maconochiei*, *H*_o_ ranged from *H*_o_ = 0.061 in Cox Peninsula Road and to *H*_o_ = 0.071 in Bynoe 2, with a mean of *H*_o_ = 0.023. *H*_e_ ranged from *H*_e_ = 0.052 in Bynoe 2 to *H*_e_ = 0.061 in Dundee Beach 1, with a mean of *H*_e_ = 0.056 (Table 2). Like *C. armstrongii* these results indicate low levels of genetic diversity*. G*_IS_ values were not significantly different from zero and ranged from *G*_IS_ = −0.138 in Bynoe 1 to *G*_IS_ = −0.186 in the *C. maconochiei* subsp. *maconochiei* Cox Pen 3 population, with an average across all populations of *G*_IS_ = −0.153 (Table 2).

**Table 2.** Summary of population genetic statistics for populations of *Cycas maconochiei* subsp. *maconochiei* and *C. armstrongii × maconochiei*.

Population	*N*	Eff*N*	*H*_O_	*H*_e_	*G*_IS_
CM Wagait Beach	1.177	1.073	0.064	0.056	−0.146
CM Belyuen	1.230	1.075	0.066	0.057	−0.145
CM Cox Peninsula Road	1.231	1.068	0.061	0.053	−0.156
Dundee Beach	1.284	1.079	0.071	0.061	−0.162
Dundee Downs	1.271	1.074	0.066	0.057	−0.150
Bynoe 2	1.271	1.069	0.061	0.052	−0.186
Bynoe 1	1.231	1.061	0.066	0.058	−0.138
Dundee Forest	1.258	1.075	0.062	0.054	−0.145
Total	1.244	1.071	0.064	0.056	−0.153
*Leviathan Creek	1.240	1.070	0.023	0.020	−0.130

^*^Leviathan Creek = *C. armstrongii × maconochiei.*

For *Cycas armstrongii × maconochiei*, *H*_o_ was 0.023, and *H*_e_ was 0.020, indicating low levels of genetic diversity. *G*_IS_ = −0.130 was not significantly different from zero (Table 2). Regional pairwise *F*_ST_ values (Table 3) revealed the lowest levels of genetic differentiation between the Darwin Coastal and Pine Creek (*F*_ST_ = 0.006, *P* > 0.05), and the highest differentiation between Cobourg and Darwin Coastal (*F*_ST_ = 0.05, *P* < 0.01). For detailed population-level estimates and significance levels, see Supplementary Data Table S3.

**Table 3.** Pairwise distance-based *F*_ST_ matrix of *Cycas armstrongii*, *C. maconochiei* subsp. *maconochiei* and *C. armstrongii × maconochiei*.

	Tiwi	Cobourg	Darwin Coastal	Pine Creek
Tiwi	–	*	*	*
Cobourg	0.014	–	*	*
Darwin Coastal	0.036	0.05	–	*
Pine Creek	0.04	0.049	0.006	–

*F*_ST_ was tested between IBRA7-defined subregions, and significance was tested with 999 permutations.

* = significant.

The AMOVA (Table 3 and Supplementary Data Table S2) was used to consider hierarchical genetic structure by IBRA7-defined region and species. By region, the results demonstrated that 86.36 % of the genetic variation is retained within individuals, with 9.26 % between individuals, 2.41 % between populations and 1.97 % between regions. Comparably, by species, the results showed that 86.39 % of the genetic variation is retained within individuals, with 9.09 % between individuals, 2.70 % between populations and 1.81 % between species. This indicates that there is a slightly higher differentiation between regions defined by geographic location than there is between species. The Mantel test identified a significant positive correlation between pairwise *F*_ST_ values and geographic distances across all populations (*r* = 0.606, *P* < 0.0001), indicating a pattern of isolation by distance (Supplementary Data Fig. S2). This result suggests that spatial separation among populations is associated with increased genetic differentiation.

### Population structure

**Fig. 2.**
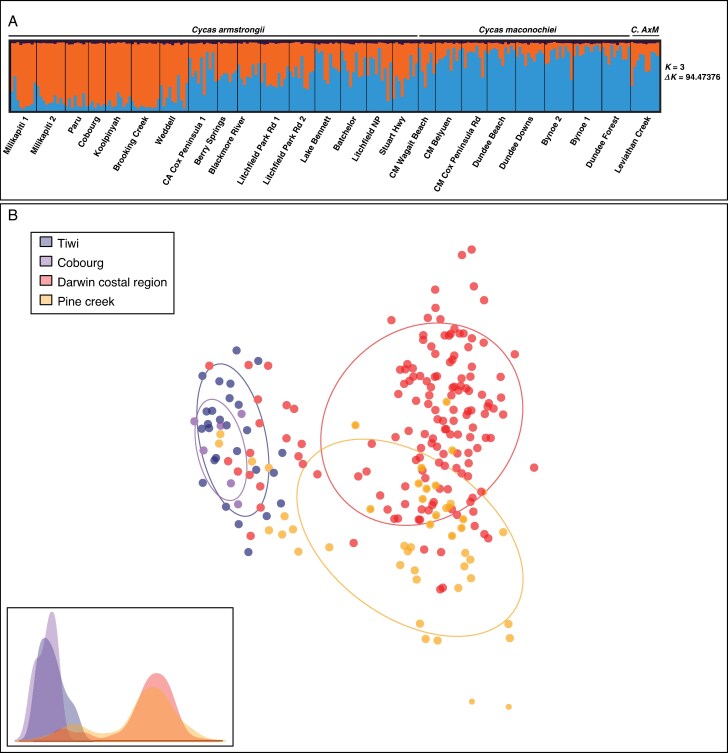
Population genetic structure for *Cycas armstrongii*, *C. maconochiei* subsp. *maconochiei* and *C. armstrongii × maconochiei*. (*C. AxM*) (A) STRUCTURE plots represent 226 samples from 25 populations of *C. armstrongii* (*n* = 142) and *C. maconochiei* subsp. *maconochiei* (*n* = 74), and a population of suspected hybrid of *C. armstrongii × maconochiei* (*n* = 10). The most likely number of genetic groups for the species was *K* = 3 and Δ*K* = 94.47376, indicating three genetic groups. However, as the third cluster is uniformly and minimally represented across all individuals, we considered *K* = 2 as the biologically meaningful number of genetic groups. (B) DAPC shows genetic differentiation between *C. armstrongii* and *C. maconochiei* ssp. *maconochiei* and a single hybrid population. The plot represents all populations within the four IBRA7 subregions, in the Northern Territory, Australia, where the taxa occur. DAPC is a summary of 21 PCs for the PCAs with three discriminant functions and a proportion of conserved variance of 0.422. The inset represents a histogram of the first axis to support the DAPC.

**Fig. 3.**
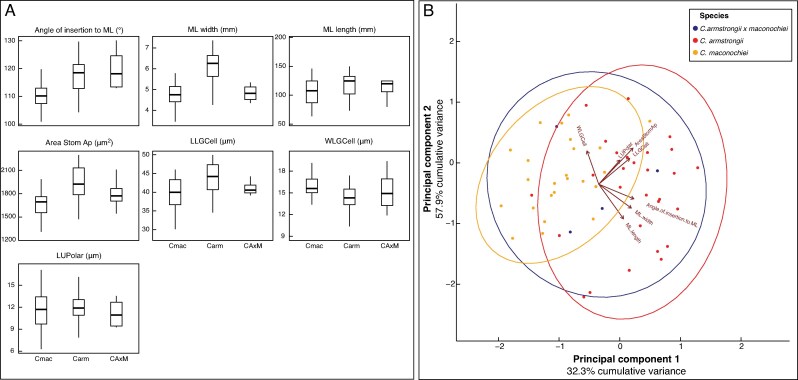
Visualization of seven morphological and micromorphological traits between *Cycas armstrongii* (Carm; *n* = 41), *C. maconochiei* ssp. *maconochiei* (Cmac; *n* = 36) and a hybrid population (CAxM; *n* = 4). (A) Box plots visualizing morphological traits of taxa showing the median, interquartile range and range of character values for each taxon. (B) PCA based on seven morphological and micromorphological traits between taxa. Angle of insertion to ML, angle of insertion of the median leaflet to the rachis; ML width, width of the median leaflet; ML length, length of the median leaflet; Area Stom Ap, area of the stomatal apparatus; LLGCell, length of leaf guard cell; WLGCell, width of left guard cell; LUpolar, length of upper polar extension.

The most likely number of distinct genetic groups was identified as *K* = 3, based on the highest Δ*K* value of 94.47 using the Evanno method, and a high posterior probability Pr[*X*|*K*] according to the Pritchard method (Pritchard *et al.*, 2000; Evanno *et al.*, 2005; Li and Liu, 2018; Fig. 2A; Supplementary Data Table S4; Supplementary Data Fig. S1). Although the methods suggested *K* = 3, the STRUCTURE plots show that the third cluster is uniformly and minimally represented across all individuals (Fig. 3A). This pattern, which is repeated in all *K*s, suggests it is an artefact of model overfitting rather than a biologically meaningful genetic group. Therefore, we consider *K* = 2 to represent the true underlying population structure, supported by distinct genetic differentiation and interpretability. The DAPC analysis also supports *K* = 2. Interestingly, neither *C. armstrongii*, *C. maconochiei* subsp. *maconochiei* nor their hybrid *C. armstrongii × maconochiei* formed distinct genetic clusters, and only minimal differentiation between species was observed (Fig. 2A). The DAPC identified two genetic groups based on a summary of 21 principal component (PC) axes, utilizing three discriminant functions, with a proportion of conserved variance of 0.422. These groups correspond to three of the four IBRA7-defined subregions (Fig. 2B). Additionally, the results revealed no genetic differentiation between the species, consistent with the STRUCTURE analysis (Fig. 2A).

### Morphology

Overall, the morphological characters showing the highest variation included median leaflet (ML) width, particularly in *C. armstrongii*, the area of the stomatal apparatus, the angle of leaflet insertion to the rachis, and the length of the left guard cell (LLGCell) (Supplementary Data Table S5). Pairwise *t*-tests comparing *C. armstrongii*, *C. maconochiei* subsp. *maconochiei* and their putative hybrid (*C. armstrongii* × *maconochiei*) indicated significant differentiation, primarily driven by micromorphological traits (Fig. 3A, Supplementary Data Tables S5 and S6). Significant differences between *C. armstrongii* and *C. maconochiei* subsp. *maconochiei* were detected in the angle of ML insertion (*P* < 0.001), ML width (*P* < 0.001), stomatal apparatus area (*P* = 0.001), width of the left guard cell (WLGCell; *P* = 0.002), and the length of the upper polar extension (LLGCell; *P* = 0.005). In contrast, only ML width differed significantly between *C. armstrongii* and the putative hybrid (*P* = 0.003), and no characters differed significantly between *C. maconochiei* subsp. *maconochiei* and the hybrid. These results suggest that *C. armstrongii* × *maconochiei* represents a morphological intermediate between the two extremes, rather than a distinct hybrid form.

The box plots used to visualize seven morphological characters across taxa (Fig. 3A), revealing substantial overlap in character distributions among taxa for most characters, although *C. maconochiei* exhibited a slightly greater range in leaflet width and length. The putative hybrid group displayed intermediate values for several characters, especially in leaflet width and the area of the stomatal apparatus, consistent with morphological intermediary in the charciters that define each taxa. However, none of the characters showed complete differentiation between taxa, highlighting the morphological plasticity.

Principal component analysis (PCA; Fig. 3B), based on seven morphological and micromorphological characters, did not reveal distinct groupings among the three taxa. However, *C. armstrongii* samples generally clustered towards the positive end of PC1, while *C. maconochiei* subsp. *maconochiei* clustered towards the negative end, with *C. armstrongii* × *maconochiei* samples occupying an intermediate position. This pattern was primarily driven by variation in leaflet width, stomatal apparatus area and leaflet insertion angle, which contributed most strongly to PC1. The relative influence of these traits varied across individuals, reflecting a continuum of morphological variation rather than discrete trait character states.

The morphological and climate correlation analysis revealed several statistically significant associations (Supplementary Data Fig. S3). Leaflet micromorphological characters, including length of the left guard cell (LLGCell) and width of the left guard cell (WLGCell), exhibited moderate correlations with temperature. Notably, LLGCell length was negatively correlated with annual mean temperature (*r* = −0.36, *P* < 0.05), and WLGCell width was positively correlated with precipitation seasonality (*r* = 0.41, *P* < 0.05). Overall, the results suggest that climatic factors have subtle yet significant influences on stomatal and leaflet morphological traits across the sampled populations.

## DISCUSSION

This study assessed the genetic diversity, differentiation and population structure of two widely distributed cycad taxa in the Northern Territory of Australia: *C. armstrongii* and *C. maconochiei* subsp. *maconochiei*. Our findings revealed low levels of genetic diversity in both species, raising concerns about their adaptive potential, particularly in the context of ongoing, threat-driven population declines. Additionally, populations exhibited limited genetic and morphological differentiation, with no clear population structure aligning with currently recognized species boundaries. This suggests that both taxa likely form a continuum of a single morphologically plastic species, calling into question the recognition of the interspecific hybrid, *C. armstrongii × maconochiei*. Our findings provide a basis for establishing conservation priorities, managing population-level threats and identifying future conservation management units for at-risk populations.

### Population genetic diversity

Populations of *C. armstrongii* and *C. maconochiei* subsp. *maconochiei* exhibit low levels of genetic diversity, suggesting limited adaptive potential within populations (Reed and Frankham, 2003). This may reduce their capacity to respond to environmental stressors, resist pathogens or recover from demographic declines (Toczydlowski and Waller, 2019), making them increasingly vulnerable to ongoing threats such as invasive species, land clearing and habitat degradation (IUCN, 2025). These challenges underscore the importance of monitoring population reductions and identifying conservation priorities, particularly for species with small or fragmented populations (Harrisson *et al.*, 2014). Comparable patterns have been observed in *Pinus yunnanensis* var. *tenuifolia*, where low heterozygosity and inbreeding were associated with reduced seed set and germination (Li *et al.*, 2024). However, few comparable data exist for cycads, highlighting the urgent need to fill knowledge gaps around genetic diversity in this imperilled plant group.

While no published population genetic data exist for *C. maconochiei* subsp. *maconochiei*, Liddle (2009) modelled habitat loss scenarios for *C. armstrongii*, which contributed to its listing as Vulnerable under Northern Territory legislation. Similarly, comparative data from other Australian cycads show variable levels of genetic diversity. For example, based on RADseq data, populations of *C. calcicola* exhibited heterozygosity levels twice as high as those of the target species in this study, although overall heterozygosity was still low (*H*_e_ = 0.095; Clugston *et al.*, 2022). In contrast, *C. megacarpa* populations showed much higher diversity when applying microsatellite makers (*H*_e_ = 0.269; James *et al.*, 2018). However, these values are not directly comparable due to differences in marker types, with microsatellites often overestimating diversity relative to SNPs (Fischer *et al.*, 2017; Hodel *et al.*, 2017; Lemopoulos *et al.*, 2019; Sunde *et al.*, 2020). Consequently, *C. megacarpa* may have lower diversity if assessed using genomic methods.

The low genetic diversity observed in both *C. armstrongii* and *C. maconochiei* subsp. *maconochiei* likely reflects a combination of evolutionary and demographic factors, including limited effective population sizes. Low levels of genetic diversity, despite the absence of evidence of inbreeding, combined with slow growth rates, longevity and low mutation rates, may indicate a historical bottleneck. This implies that any increase in genetic diversity is likely to occur only over extended time periods. A comparable case is seen in *C. segmentifida*, where long generation times and historical population contractions were found to contribute to the slow accumulation of genetic diversity (Feng *et al.*, 2017). While low rates of sexual reproduction have been proposed as a contributing factor to reduced genetic diversity in cycads, this is unlikely to be the case in these two species (Ornduff, 1991). Additionally, the slow generation times typical of cycads (Griffith *et al.*, 2015) may further constrain mutation rates and limit short-term adaptability. Gymnosperms have experienced high extinction rates and more recent radiations (Nagalingum *et al.*, 2011), which may explain their generally lower genetic diversity compared with angiosperms (Crisp and Cook, 2011).

### Population differentiation and geographic distribution

Analysis of molecular variance for *C. armstrongii* and *C. maconochiei* subsp. *maconochiei* showed most variation was within individuals (Supplementary Data Table S2, 86.39 %), followed by variation among individuals (9.09 %). The level of variance among populations for each species was low (2.7 %), and differentiation was lowest when testing between *C. armstrongii* and *C. maconochiei* subsp. *maconochiei* (1.81 %), suggesting minimal genetic differences between the two taxa. These findings suggest at least historical gene flow and connectivity among populations. Similar trends have also been seen in *Macrozamia riedlei* (Byrne and James, 1991) and *Podocarpus elatus* (Mrillick *et al.*, 2011), where populations show low genetic differentiation throughout their ranges due to historical gene flow and effective dispersal strategies. Similar patterns have been noted worldwide, including *Ginkgo biloba*, which exhibits low population differentiation due to long-distance seed dispersal and human-assisted movement (Gong *et al.*, 2008), and *Pinus sylvestris*, which displays extensive gene flow across Eurasia despite differing environmental conditions (Pyhäjärvi *et al.*, 2007). These cases emphasize the role of historical connectivity and life-history traits on the genetic structure of gymnosperms both in Australia and globally. Increased levels of pairwise genetic differentiation (Table 3) were observed between all mainland populations and the Tiwi Cobourg population (Fig. 2B). This is somewhat expected due to the geographic distance between populations within these regions, especially between populations on the Tiwi Islands (which fall ∼80 km offshore) and the mainland (90 and 165 km), which would severely limit gene flow and correlates with isolation by distance (Fig. 1C).

Genetic differentiation was lowest between populations at Darwin Coastal and Pine Creek, indicative of higher gene flow correlating with reduced geographic distance. Similar patterns were found in populations of *Clarkia pulchella* where geographic distance directly correlated with genetic distance and defined the population’s structure (Bontrager and Angert, 2018). Although there are no distinct differences in the genetic structure of the populations (Fig. 2A), the Mantel test revealed significant isolation by distance (*r* = 0.606, *P* < 0.0001, Supplementary Data Fig. S2), indicating that the greater geographic distance between mainland Australia and the Tiwi Islands (Fig. 1C) correlated with greater genetic differences (Supplementary Data Table S6). This admixture and close genetic relationship imply that managed gene flow, such as translocating individuals (seedlings) or seeds between populations, may be a viable conservation strategy in geographically close populations. This can improve the adaptive potential of small and isolated populations (Frankham, 2015). Implementing genetic rescue strategies, informed by genomic data, can thus be instrumental in maintaining the long-term viability of these cycad populations. Genetic rescue has been successfully applied in various taxa, including *Populus* (Aitken and Whitlock, 2013), *Arabidopsis* (Pickup *et al.*, 2012) and *Silene* (Willi *et al.*, 2007), where gene flow introduced from genetically distinct populations has been shown to increase genetic fitness and resilience in populations. These examples highlight the potential of carefully planned interventions to mitigate genetic erosion and reduce extinction risk in fragmented plant populations.

Buoyancy aids found in the seeds of some *Cycas* species, such as *C. rumphii*, have contributed to shaping their geographic distribution (Dehgan and Yuen, 1983; Nadarajan *et al.*, 2018; Liu *et al.*, 2021). However, for species lacking such adaptations, including *C. armstrongii* and *C. maconochiei* s.l., long-distance seed dispersal may be significantly constrained. The presence of populations on the Tiwi Islands is especially intriguing, implying historical seed movement between the islands and mainland Australia. One probable mechanism is human-mediated dispersal, considering indigenous Australians have occupied these lands for at least 65 000 years (Morrison *et al.*, 2023). There is also evidence of seed processing and consumption of *C. armstrongii* by Aboriginal communities in the Darwin region (Beck, 1992). Unprocessed seeds of both species were probably transported as a food source between the mainland and the Tiwi Islands. Similar patterns have been observed in other plant groups where indigenous seed movement has shaped species distributions and plant community composition (Beaton, 1982; Hynes and Chase, 1982; Cooke *et al.*, 2024; Fahey *et al.*, 2024). Unlike *C. calcicola*, which has a more restricted range but exhibits greater population differentiation (Clugston *et al.*, 2022; Fig. 2), *C. armstrongii* and *C. maconochiei* subsp. *maconochiei* show comparatively lower genetic differentiation. One possible factor is seed size: *C. calcicola* produces seeds up to 10 mm smaller than those of the other species examined (Hill, 1996). These smaller seeds may have been less desirable to indigenous Australians for consumption or use, potentially reducing human-mediated dispersal and increasing population isolation (Beck, 1992).

### Differences between taxa and the purported hybrid population

Our results revealed minimal genetic differentiation and structure between *C. armstrongii* and *C. maconochiei* subsp. *maconochiei*, with most genetic variation found within populations rather than between taxa. This lack of structure parallels the occurrence of morphologically intermediate individuals and raises questions about the genetic distinctiveness of *C. maconochiei* subsp. *maconochiei* populations (I. Cowie, 2018, Northern Territory Department of Lands, Planning and Environment, Australia,pers. comm.). While significant morphological differences exist, cycads are reported to be morphologically plastic (Newell, 1985; Woodenberg *et al.*, 2019), including *Cycas* (Hill, 2004). For example, Chang *et al.* (2023) found limited genetic and morphological differentiation between *C. revoluta* and *C. taitungensis*, concluding that neither genetic nor morphological evidence supported their recognition as distinct species. Similarly, He *et al.* (2023) reported low genetic divergence among several *Cycas* taxa in south-western China, despite their current classification. Together, these studies and our findings suggest that the number of genetically distinct *Cycas* species may be overestimated (Hsiao and Oberprieler, 2024).

Although hybridization is well documented in cycads (Chamberlain, 1926; Norstog, 1990; Calonje *et al.*, 2011) and could theoretically explain the morphological intermediacy observed in the purported *C. armstrongii × maconochiei* hybrid population, our results provide no genetic support for recent admixture between this population and either putative lineage. In contrast to cases where hybrid taxa display noticeable genetic structures, they often exhibit a mosaic of morphological characteristics from both parent species rather than a continuous spectrum (e.g. *Carex* spp., Schmidt *et al.*, 2018; *Calotropis* spp., Muriira *et al.*, 2018). *Cycas armstrongii* and *C. maconochiei* subsp. *maconochiei* show genetic unity, which implies a lack of reproductive isolation and complicates the current classification of these species.

While micromorphological traits, including cuticle features, can effectively differentiate some cycad genera (e.g. *Ceratozamia*, *Dioon* and *Encephalartos*), they are not easily distinguishable in the field. They can be affected by environmental factors, as shown in Podocarpaceae species (Clugston *et al.*, 2017). The morphological findings partially align with the genetic data (Fig. 3), as individuals from the putative hybrid population did not exhibit distinct traits but instead fell along a morphological continuum between the two taxa. Nonetheless, five of the seven measured traits showed significant differences between *C. armstrongii* and *C. maconochiei* subsp. *maconochiei*, indicating clear morphological differentiation between the two morphotypes. While many leaflet traits in cycads are known to have limited diagnostic power compared with characters such as leaflet recurve and cataphyll morphology (Hill, 1996), the leaflet trait data used here effectively capture patterns of morphological overlap and potential intermediacy. This trend has been observed in other plant groups, such as *Platanthera* (Bateman *et al.*, 2013) and *Vriesea* (Neri *et al.*, 2018), reinforcing the notion that *C. armstrongii* and *C. maconochiei* subsp. *maconochiei* represent a single, morphologically diverse taxon, rather than two genetically isolated lineages housing an interspecific hybrid population.

From a climatic perspective, four of the seven traits show significant positive or negative correlations with climate variables (Supplementary Data Fig. S3), suggesting they are environmentally responsive and likely represent adaptations to local microclimates. This may explain the observed morphological differences between *C. armstrongii* and *C. maconochiei* subsp. *maconochiei*, despite limited genetic differentiation. A comparable pattern has been observed in Podocarpaceae, where plants growing in different climatic zones exhibited significant morphological differences between wild and cultivated individuals, highlighting the role of environmental adaptation (Clugston *et al.*, 2017). These findings highlight the need to refine species boundaries in *C. armstrongii* s.l., ensuring conservation efforts focus on evolutionarily and ecologically meaningful units (Coates *et al.*, 2018). However, a key sampling gap remains in the Wadeye/Fitzmaurice River region, recognized as *C. maconochiei* subsp. *maconochiei* (*sensu* Hill, 1996), which is spatially isolated and potentially morphologically distinct. Future genetic and demographic surveys in this region will be crucial for clarifying its relationship to other populations and ensuring appropriate taxonomic resolution.

### Conservation implications and research priorities

Taxonomic inflation has profound implications for conservation (Isaac *et al.*, 2004), and the overreliance on morphological traits may lead to species misidentification, resulting in the mismanagement of biologically meaningful management units (Swarts *et al.*, 2014). More critically, scarce conservation resources risk being diverted towards maintaining artificial species boundaries, rather than supporting ecologically or genetically significant populations (Gordon *et al.*, 2020). In this context, conserving what may represent a morphological variant rather than a distinct species could misdirect conservation investments. For *C. armstrongii* and *C. maconochiei* s.l., this study supports a taxonomic revaluation to ensure that conservation priorities reflect true biological diversity.

Several populations of *C. armstrongii* and *C. maconochiei* subsp. *maconochiei*, particularly those from the Tiwi Islands (Milikapiti 1, Paru, Koolpinyah), Belyuen and Litchfield Park Road, exhibited very low genetic diversity (*H*_e_ < 0.030), which may correlate with reduced adaptive potential and heightened extinction risk (Ottewell *et al.*, 2016). Similar patterns have been reported in *Cycas segmentifida*, where geographic isolation and habitat fragmentation contributed to genetic divergence and reduced reproductive connectivity (Feng *et al.*, 2017). We therefore recommend prioritizing these populations for detailed demographic assessment and recognition as distinct ecological conservation management units (CMUs).

Although CMUs are not formal legal entities under the Environment Protection and Biodiversity Conservation Act (EPBC Act; Australian Government, 1999), they are increasingly recognized as essential for prioritizing conservation actions at the population level (Doyle *et al.*, 2025). Genetically or demographically unique populations, especially those with ecological or cultural importance, have been prioritized for protection even in the absence of formal taxonomic status (Coates *et al.*, 2018; Palsbøll *et al.*, 2007). For instance, Bradbury *et al.* (2023) identified eco-geographically distinct CMUs in *Conospermum caeruleum* despite minimal genetic divergence. CMUs can inform the development of recovery plans and ensure that deprived populations receive tailored conservation attention.

Recent examples demonstrate the feasibility and value of applying genomics in population-level conservation. For instance, in Australia, the New South Wales *Saving our Species* programme has integrated genomics into a range of conservation projects, including efforts to clarify taxonomy, prioritize monitoring, guide translocations and establish *ex situ* collections. Despite initial concerns over cost, genomic work accounted for <10 % of total conservation investment and is becoming increasingly more cost-effective relative to other recovery actions (Rossetto et al., 2021). This case underscores the utility of incorporating genomics into conservation planning to optimize long-term outcomes and success rates.

Given the close genetic relationship among many populations, conservation interventions such as assisted gene flow, e.g. translocating seedlings or seeds, represent a promising strategy to enhance diversity, reduce inbreeding and improve adaptive capacity in small or isolated populations (Frankham, 2015). Van Rossum and Hardy (2022) demonstrated the success of such strategies in restoring genetically poor populations. We recommend prioritizing conservation actions for populations that are small, geographically restricted or genetically deprived. These populations face elevated threats from habitat transformation, altered fire regimes and invasive species (Department of Climate Change, Energy, the Environment and Water, 2021). Where population declines are observed or anticipated, establishing genetically informed *ex situ* assurance collections should also be considered.

While RADseq revealed broad-scale genetic structure, it is less suited for fine-scale resolution within the *C. armstrongii* s.l. group due to limitations such as low genome coverage and poor reproducibility (Paris *et al.*, 2017; Shafer *et al.*, 2017; McCartney-Melstad *et al.*, 2019). As sequencing technologies advance, continuing to rely on RADseq may constrain comparative analyses. In contrast, emerging genomic resources for cycads, including a draft genome and transcriptomes (Liu *et al.*, 2022*a*, *b*), support a shift to more integrative methods. Target capture sequencing provides higher resolution and reproducibility across studies (Heyduk *et al.*, 2016; Johnson *et al.*, 2019), particularly for long-lived taxa like cycads with conserved genomes (Hale *et al.*, 2020). Approaches such as hybrid capture or genome skimming offer scalable frameworks for generating consistent data to support conservation planning (Pezzini *et al.*, 2023). If *C. maconochiei* subsp. *maconochiei* is formally reclassified under *C. armstrongii*, this would require a reassessment of conservation status at the national level. Currently, *C. armstrongii* is listed as Vulnerable under the Northern Tertiaries TPWCA (2021), while *C. maconochiei* is listed as Least Concern, and neither species is listed under the EPBC Act. A broader taxonomic circumscription, unless paired with population-level genetic and demographic data, risks obscuring the conservation needs of small or declining populations. Standardized assessment frameworks should be used to evaluate extinction risk at both species and population levels. In particular, targeted surveys in the Wadeye/Fitzmaurice region will be essential for understanding the genetic diversity and demographic distinctiveness of populations in this undersampled part of the species’ range.

### Conclusions

*Cycas armstrongii* and *C. maconochiei* subsp. *maconochiei* are widely distributed across the Northern Territory of Australia; however, our results revealed consistently low levels of genetic diversity in both taxa, raising concerns about their long-term adaptive potential. Despite geographic separation, particularly between Tiwi Island and mainland populations, genetic differentiation among regions was minimal. Although morphological analyses showed some differentiation between taxa, these characters are likely environmentally linked, indicating the population previously regarded as a putative hybrid, most likely representing a morphological intermediate or two morphotypes.

Genetic and morphological data therefore suggest that the sampled populations represent a single, morphologically variable species rather than two distinct taxa. However, we caution against a formal taxonomic revision at this time, as sampling of *C. maconochiei* was incomplete, particularly as one locality of *C. maconochiei* subsp. *maconochiei* was not included, and no populations attributable to *C. maconochiei* subsp. *viridis* or *C. maconochiei* subsp. *lanata* were sampled (*sensu* Hill, 1996). Broader sampling across the full geographic range of *C. maconochiei* is therefore essential to evaluate species boundaries more robustly.

Expanding the circumscription of *C. armstrongii* based on limited evidence may artificially reduce the species’ level of threat due to the increased estimates of extent of occurrence and area of occupancy, potentially obscuring the vulnerability of genetically depauperate or geographically isolated populations. We recommend that any future taxonomic or conservation status changes be based on comprehensive sampling and evaluated using standardized extinction risk frameworks. In the meantime, populations exhibiting low genetic diversity or exposure to localized threats should be treated as priority CMUs, guiding targeted conservation and recovery actions. This approach will help ensure that management strategies reflect both evolutionary patterns and ecological vulnerability.

## Supplementary Material

mcaf276_Supplementary_Data

## SUPPLEMENTARY DATA

Supplementary data are available at *Annals of Botany* online and consist of the following. Table S1: summary of samples collected from natural populations of *Cycas armstrongii* complex in Northern Territory, Australia. Table S2: analysis of molecular variance (AMOVA) for the *Cycas armstrongii* complex. Table S3: pairwise distance-based *F*_ST_ matrix for populations *Cycas armstrongii*, *Cycas maconochiei* ssp. *maconochiei* and *C. armstrongii × maconochiei*. Table S4: StructureSelector results to help support the number of genetic groups from STRUCTURE analysis. Table S5: summary of morphological character measurements for *C. armstrongii*, *C. maconochiei* subsp. *maconochiei* and *C. armstrongii × maconochiei*. Table S6: pairwise *t*-test showing micromorphological/macromorphological variation of seven characters between *C. armstrongii*, *C. maconochiei* subsp. *maconochiei* and *C. armstrongii × maconochiei*. Table S7: complete sample metadata. Figure S1: *K* plots output from StructureSelector indicated *K* = 3 as the most probable (Δ*K* = 94.47) number of genetic clusters among all sampled taxa representing *Cycas armstrongii*, *Cycas maconochiei* ssp. *maconochiei* and the suspected putative hybrid population *C. armstrongii × maconochiei*. Figure S2: geographic and genetic distances between populations of *Cycas armstrongii* and *C. maconochiei* ssp. *maconochiei*. The pairwise genetic differentiation (*F*_ST_) was plotted against the geographic distance (km) between populations, with the red dashed line representing the linear regression fit. A Mantel test showed a significant positive correlation (*r*^2^ = 0.606, *P* < 0.0001), indicating a pattern of isolation by distance. Figure S3: heat map displaying Pearson correlation coefficients (*r* values) between seven morphological traits and six climate variables for the *Cycas armstrongii* group. Blue and red cells denote negative and positive correlations, respectively. Asterisks (*) mark significant correlations (*P* < 0.05). Angle of insertion to ML = angle at which the median leaflet inserts to the rachis (°); ML width = width of the median leaflet (mm); ML length = length of the median leaflet (mm); AreaStomAp = area of the stomatal apparatus (μm²); LLGCell = length of the left guard cell (μm); WLGCell = width of the left guard cell (μm); LUPolar = length of the upper polar extension (μm).

## AI ASSISTANCE

AI was not used in the writing of this manuscript. However, AI was used in the editing process to help with grammar and spelling.

## DATA ACCESSIBILITY AND BENEFIT-SHARING

Raw sequence reads and sample metadata are deposited in the SRA (BioProject: PRJNA1364755.
