# Supplementary material for: Low genetic differentiation among morphologically distinct Cycas species informs the delineation of conservation management units
Source: Ann Bot. 2025 Nov 13;137(2):415–30. doi: 10.1093/aob/mcaf276 (PMC12823241; doi:10.1093/aob/mcaf276)
Supplement: mcaf276_Supplementary_Data [file mcaf276_supplementary_data.zip › Supplementary Table 1.docx]

**Supplementary Table 1. Summary of samples collected from natural populations of *Cycas armstrongii* complex in Northern Territory, Australia*.*** The table defines the stratification levels used for discriminant analysis and AMOVA. Species = The identification assigned during collection, Region and Sub-Region = define the regions and sub-regions for the Interim Biogeographic Regionalisation for Australia (IBRA7) from Australia's Strategy for the National Reserve System (<http://www.environment.gov.au/land/nrs/science/ibra>). **Population** = individual populations where collections were carried out and **No. samples** = the total number of samples collected from each population.

| **Species** | **IBRA Region** | **IBRA Subregion** | **Population** | **No. Samples** |
| --- | --- | --- | --- | --- |
| *C. arm X mac* | DAC | DAC01 | Leviathan Creek | 10 |
| *C. maconochiei* | Darwin Coastal  (DAC) | Darwin Coastal  (DAC01) | Dundee Forest | 7 |
|  |  |  | Bynoe 1 | 10 |
|  |  |  | Bynoe 2 | 10 |
|  |  |  | Dundee Downs | 10 |
|  |  |  | Dundee Beach 1 | 10 |
|  |  |  | CM Cox Peninsula Rd | 9 |
|  |  |  | CM Belyuen | 9 |
|  |  |  | CM Wagait Beach | 6 |
| *C. armstrongii* | Darwin Coastal  (DAC) | Darwin Coastal  (DAC01) | Litchfield Park Rd 1 | 10 |
|  |  |  | Blackmore River | 8 |
|  |  |  | Berry Springs | 7 |
|  |  |  | Cox Peninsula Rd | 10 |
|  |  |  | Weddell | 10 |
|  |  |  | Brooking Creek | 10 |
|  |  |  | Koolpinyah | 10 |
|  | Pine Creek  (PCK) | Pine Creek  (PCK01) | Stuart Highway | 8 |
|  |  |  | Litchfield NP 1 | 9 |
|  |  |  | Batchelor 1 | 9 |
|  |  |  | Lake Bennett | 10 |
|  |  |  | Litchfield Park Rd 2 | 9 |
|  | Tiwi Coburg  (TIW) | Tiwi  (TIW01) | Milikapiti 1 | 9 |
|  |  |  | Milikapiti 2 | 10 |
|  |  |  | Paru | 8 |
|  |  | Cobourg  (TIW02) | Cobourg | 6 |
