# Supplementary material for: Low genetic differentiation among morphologically distinct Cycas species informs the delineation of conservation management units
Source: Ann Bot. 2025 Nov 13;137(2):415–30. doi: 10.1093/aob/mcaf276 (PMC12823241; doi:10.1093/aob/mcaf276)
Supplement: mcaf276_Supplementary_Data [file mcaf276_supplementary_data.zip › Supplementary Table 2.docx]

**Supplementary Table 2 Analysis of Molecular Variance for the *Cycas armstrongii* Complex.** AMOVA analysis results between all populations, regions and species for *Cycas armstrongii* and *Cycas maconochiei* ssp. *maconochiei*. Differences between areas = genetic variation between populations within IBRA7 defined regions, Between populations = the degree of genetic variation between populations, between individuals = the degree of genetic variation between individuals nested in populations and Within individuals = the genetic variation within individuals within populations. Between species = the degree of genetic variation among species, including *Cycas armstrongii*, *Cycas maconochiei* ssp. *maconochiei* and the suspected putative hybrid population *C. armstrongii* x *maconochiei*.

| **Differences between region** | | | | **Differences between species** | | | |
| --- | --- | --- | --- | --- | --- | --- | --- |
|  | **Df** | **Sum Sq** | **Mean Sq** |  | **Df** | **Sum Sq** | **Mean Sq** |
| Between region | 3 | 758.65 | 252.88 | Between species | 2 | 610.78 | 305.39 |
| Between population | 19 | 2474.74 | 130.25 | Between population | 22 | 2860.27 | 130.01 |
| Between individuals | 203 | 18078.23 | 89.06 | Between individuals | 201 | 17840.57 | 88.76 |
| Within individuals | 226 | 16573.15 | 73.33 | Within individuals | 226 | 16573.15 | 73.33 |
| Total | 451 | 37884.77 | 84.00 | Total | 451 | 37884.77 | 84.00 |
| **Variance components:** | **Sigma** | **%** |  | **Variance components:** | **Sigma** | **%** |  |
| Between Region | 1.67 | 1.97 |  | Between Species | 1.53 | 1.81 |  |
| Between Population | 2.05 | 2.41 |  | Between Population | 2.30 | 2.70 |  |
| Between individuals | 7.86 | 9.26 |  | Between individuals | 7.71 | 9.09 |  |
| Within individuals | 73.33 | 86.36 |  | Within individuals | 73.33 | 86.39 |  |
| Total variations | 84.91 | 100 |  | Total variations | 84.88 | 100 |  |
| **Phi:** |  |  |  | **Phi:** |  |  |  |
| Phi-samples-total | 0.1364 |  |  | Phi-samples-total | 0.1360 |  |  |
| Phi-samples-Population | 0.0968 |  |  | Phi-samples-Population | 0.0951 |  |  |
| Phi-Population-Region | 0.0246 |  |  | Phi-Population-Region | 0.0275 |  |  |
| Phi-Region-total | 0.0197 |  |  | Phi-Region-total | 0.0181 |  |  |
