# Supplementary material for: Low genetic differentiation among morphologically distinct Cycas species informs the delineation of conservation management units
Source: Ann Bot. 2025 Nov 13;137(2):415–30. doi: 10.1093/aob/mcaf276 (PMC12823241; doi:10.1093/aob/mcaf276)
Supplement: mcaf276_Supplementary_Data [file mcaf276_supplementary_data.zip › Supplementary Table 3.docx]

**Supplementary Table 3 Pairwise distance-based *F*_st_ matrix for populations *Cycas armstrongii*, *Cycas maconochiei* ssp. *maconochiei* and *C. armstrongii* x *maconochiei***. Values derived from AMOVA analysis. Significance was tested with 999 permutations. See Table 1 for population abbreviations.

| **Region** | **Tiwi** | | | **Cobourg** | **Darwin Costal Region** | | | | | | **Pine Creek** | | | | | | **Darwin Costal Region** | | | | | | | | |
| --- | --- | --- | --- | --- | --- | --- | --- | --- | --- | --- | --- | --- | --- | --- | --- | --- | --- | --- | --- | --- | --- | --- | --- | --- | --- |
| **Population** | **Milikapiti 1** | **Milikapiti 2** | **Paru** | **Cobourg** | **Koolpinyah** | **Brooking Creek** | **Weddell** | **Cox Peninsula Rd** | **Berry Springs** | **Blackmore River** | **Litchfield Park Road 1** | **Litchfield Park Road 2** | **Lake Bennett** | **Batchelor 1** | **Litchfield**  **NP 1** | **Stuart Highway** | **CM Blackmore River** | **CM Berry Springs** | **CM Cox Peninsula Rd** | **Dundee Beach** | **Dundee Downs** | **Bynoe 2** | **Bynoe 1** | **Dundee Forest** | **Lenathan Creek** |
| **Species** | ***C. armstrongii*** | | | | | | | | | | | | | | | | ***C. maconochiei*** | | | | | | | | **Hybrid** |
| **Milikapiti 1** | -- | 0.061 | 0.606 | * | 0.305 | ** | ** | ** | ** | ** | 0.172 | * | ** | ** | ** | ** | ** | ** | ** | ** | ** | ** | ** | ** | ** |
| **Milikapiti 2** | 0.012 | -- | 0.095 | 0.123 | * | ** | ** | ** | ** | ** | ** | * | ** | ** | ** | ** | ** | ** | ** | ** | ** | ** | ** | ** | ** |
| **Paru** | -0.002 | 0.01 | -- | ** | 0.345 | ** | ** | ** | ** | ** | 0.14 | * | ** | ** | ** | ** | ** | ** | ** | ** | ** | ** | ** | ** | ** |
| **Cobourg** | 0.017 | 0.013 | 0.024 | -- | 0.153 | ** | ** | ** | ** | ** | ** | 0.447 | ** | ** | ** | ** | ** | ** | ** | ** | ** | ** | ** | ** | ** |
| **Koolpinyah** | 0.003 | 0.019 | 0.002 | 0.01 | -- | ** | ** | ** | ** | ** | * | * | ** | ** | ** | ** | ** | ** | ** | ** | ** | ** | ** | ** | ** |
| **Brooking Creek** | 0.032 | 0.04 | 0.024 | 0.068 | 0.042 | -- | 0.124 | * | 0.388 | 0.596 | ** | ** | * | 0.873 | * | 0.355 | 0.063 | * | 0.166 | ** | * | 0.073 | ** | * | * |
| **Weddell** | 0.036 | 0.051 | 0.034 | 0.074 | 0.044 | 0.006 | -- | 0.342 | 0.608 | 0.067 | ** | ** | * | 0.714 | 0.099 | 0.231 | * | ** | 0.063 | ** | ** | ** | ** | ** | ** |
| **Cox Peninsula Rd** | 0.046 | 0.062 | 0.04 | 0.084 | 0.054 | 0.012 | 0.003 | -- | * | 0.499 | ** | ** | 0.115 | 0.253 | 0.348 | 0.536 | * | ** | ** | ** | ** | ** | ** | ** | ** |
| **Berry Springs** | 0.053 | 0.07 | 0.038 | 0.107 | 0.066 | 0.002 | -0.002 | 0.015 | -- | 0.127 | ** | ** | 0.062 | 0. | 0.118 | 0.092 | ** | * | 0.08 | ** | ** | 0.094 | ** | * | ** |
| **Blackmore River** | 0.04 | 0.04 | 0.036 | 0.053 | 0.036 | -0.002 | 0.011 | -0.001 | 0.01 | -- | ** | * | 0.355 | 0.613 | 0.328 | 0.045 | * | * | 0.069 | ** | * | * | * | * | ** |
| **Litchfield Park**  **Road 1** | 0.006 | 0.02 | 0.007 | 0.029 | 0.011 | 0.034 | 0.038 | 0.057 | 0.06 | 0.032 | -- | * | ** | ** | ** | ** | ** | ** | ** | ** | ** | ** | ** | ** | ** |
| **Litchfield Park**  **Road 2** | 0.017 | 0.017 | 0.013 | 0 | 0.015 | 0.026 | 0.038 | 0.04 | 0.047 | 0.019 | 0.015 | -- | ** | ** | ** | ** | ** | ** | ** | ** | ** | ** | ** | ** | ** |
| **Lake Bennett** | 0.043 | 0.05 | 0.027 | 0.06 | 0.038 | 0.011 | 0.016 | 0.007 | 0.011 | 0.002 | 0.037 | 0.027 | -- | * | ** | ** | ** | ** | ** | ** | ** | ** | ** | ** | ** |
| **Batchelor 1** | 0.041 | 0.05 | 0.045 | 0.07 | 0.047 | -0.007 | -0.004 | 0.004 | 0 | -0.003 | 0.037 | 0.036 | 0.014 | -- | ** | 0.398 | * | * | 0.078 | * | ** | * | ** | ** | ** |
| **Litchfield NP 1** | 0.061 | 0.062 | 0.05 | 0.097 | 0.072 | 0.009 | 0.009 | 0.002 | 0.012 | 0.004 | 0.059 | 0.04 | 0.019 | 0.015 | -- | 0.325 | * | ** | ** | ** | ** | ** | ** | ** | ** |
| **Stuart Highway** | 0.064 | 0.064 | 0.041 | 0.104 | 0.069 | 0.002 | 0.005 | -0.001 | 0.011 | 0.013 | 0.053 | 0.059 | 0.021 | 0.001 | 0.003 | -- | ** | ** | * | ** | ** | ** | ** | * | * |
| **CM Blackmore River** | 0.033 | 0.038 | 0.04 | 0.063 | 0.03 | 0.011 | 0.022 | 0.024 | 0.017 | 0.017 | 0.037 | 0.039 | 0.039 | 0.016 | 0.022 | 0.025 | -- | 0.062 | * | * | * | ** | * | * | 0.097 |
| **CM Berry Springs** | 0.047 | 0.047 | 0.034 | 0.072 | 0.059 | 0.013 | 0.016 | 0.023 | 0.013 | 0.015 | 0.053 | 0.036 | 0.024 | 0.015 | 0.022 | 0.025 | 0.014 | -- | 0 | 0.312 | 0.667 | 0.816 | 0.247 | 0.508 | 0.467 |
| **CM Cox Peninsula Rd** | 0.053 | 0.048 | 0.054 | 0.086 | 0.063 | 0.006 | 0.009 | 0.021 | 0.01 | 0.01 | 0.054 | 0.048 | 0.017 | 0.009 | 0.031 | 0.016 | 0.013 | 0 | -- | 0.091 | 0.859 | 0.136 | 0.33 | 0.529 | 0.163 |
| **Dundee Beach** | 0.058 | 0.065 | 0.049 | 0.08 | 0.077 | 0.02 | 0.037 | 0.029 | 0.022 | 0.022 | 0.069 | 0.055 | 0.032 | 0.018 | 0.024 | 0.038 | 0.016 | 0.002 | 0.007 | -- | 0.678 | * | 0.997 | 0.128 | ** |
| **Dundee Downs** | 0.051 | 0.061 | 0.051 | 0.089 | 0.072 | 0.016 | 0.039 | 0.022 | 0.023 | 0.016 | 0.063 | 0.058 | 0.026 | 0.02 | 0.025 | 0.031 | 0.021 | -0.003 | -0.007 | -0.003 | -- | 0.104 | 0.602 | 0.497 | 0.196 |
| **Bynoe 2** | 0.031 | 0.044 | 0.037 | 0.056 | 0.047 | 0.008 | 0.027 | 0.017 | 0.01 | 0.013 | 0.037 | 0.041 | 0.02 | 0.016 | 0.027 | 0.021 | 0.022 | -0.005 | 0.006 | 0.01 | 0.007 | -- | ** | 0.409 | 0.117 |
| **Bynoe 1** | 0.063 | 0.069 | 0.053 | 0.089 | 0.078 | 0.022 | 0.037 | 0.024 | 0.027 | 0.019 | 0.075 | 0.063 | 0.032 | 0.024 | 0.03 | 0.033 | 0.014 | 0.004 | 0.002 | -0.013 | -0.001 | 0.017 | -- | 0.426 | * |
| **Dundee Forest** | 0.048 | 0.049 | 0.042 | 0.076 | 0.057 | 0.011 | 0.031 | 0.027 | 0.014 | 0.013 | 0.056 | 0.05 | 0.022 | 0.02 | 0.026 | 0.029 | 0.016 | 0 | -0.001 | 0.006 | 0 | 0.001 | 0.001 | -- | 0.096 |
| **Lenathan Creek** | 0.036 | 0.042 | 0.026 | 0.051 | 0.044 | 0.011 | 0.024 | 0.025 | 0.016 | 0.018 | 0.041 | 0.036 | 0.023 | 0.019 | 0.021 | 0.018 | 0.01 | 0 | 0.005 | 0.014 | 0.005 | 0.006 | 0.012 | 0.006 | -- |
