# Supplementary material for: Low genetic differentiation among morphologically distinct Cycas species informs the delineation of conservation management units
Source: Ann Bot. 2025 Nov 13;137(2):415–30. doi: 10.1093/aob/mcaf276 (PMC12823241; doi:10.1093/aob/mcaf276)
Supplement: mcaf276_Supplementary_Data [file mcaf276_supplementary_data.zip › Supplementary Table 4.docx]

**Supplementary Table 4 StructureSelector results to help support the number of genetic groups from STRUCTURE analysis**. Data shows results from StructureSelector analysis to support population structure (Figure 2A) analysis for Δ*K* for the number of genetic groups set in STRUCTURE (*K* = 1 - 25).

| Groups - *K* | Mean LnP(*K*) | Stdev LnP(*K*) | Ln'(K) | | \|Ln''(*K*)\| | | Delta *K* |
| --- | --- | --- | --- | --- | --- | --- | --- |
| 1 | -38094.695 | 1.06251 | - | | - | | - |
| 2 | -37723.68 | 362.62347 | 371.015 | | 210.96 | | 0.58176 |
| 3 | -37141.705 | 8.04948 | 581.975 | | 760.465 | | **94.47376** |
| 4 | -37320.195 | 66.34319 | -178.49 | | 224.2 | | 3.3794 |
| 5 | -37274.485 | 222.48841 | 45.71 | | 85.465 | | 0.38413 |
| 6 | -37314.24 | 471.12732 | -39.755 | | 2182.06 | | 4.63157 |
| 7 | -39536.055 | 3229.72768 | -2221.815 | | 1840.055 | | 0.56972 |
| 8 | -39917.815 | 3000.03239 | -381.76 | | 637.81 | | 0.2126 |
| 9 | -40937.385 | 3587.51628 | -1019.57 | | 394.06 | | 0.10984 |
| 10 | -46838.17 | 6164.39434 | -4487.155 | | 7231.19 | | 0.76657 |
| 11 | -44094.135 | 4354.90496 | 2744.035 | | 883.545 | | 1.17306 |
| 12 | -42233.645 | 4083.51396 | 1860.49 | | 9060.595 | | 0.20289 |
| 13 | -49433.75 | 11856.6424 | -7200.105 | | 1045.98 | | 2.21882 |
| 14 | -55587.875 | 10799.2438 | -6154.125 | | 14052.025 | | 0.08822 |
| 15 | -47689.975 | 10775.1836 | 7897.9 | | 4309.945 | | 1.3012 |
| 16 | -44102.02 | 3632.24473 | 3587.955 | | 7886.765 | | 0.39999 |
| 17 | -48400.83 | 5470.56212 | -4298.81 | | 4507.38 | | 2.17132 |
| 18 | -48192.26 | 6104.71957 | 208.57 | | 2412.48 | | 0.82393 |
| 19 | -45571.21 | 3453.42868 | 2621.05 | | 2723.51 | | 0.39518 |
| 20 | -45673.67 | 3823.74222 | -102.46 | | 2367.745 | | 0.78864 |
| 21 | -48143.875 | 5123.89544 | -2470.205 | | 6989.38 | | 0.61922 |
| 22 | -57603.46 | 8569.35819 | -9459.585 | | 21381.82 | | 1.36408 |
| 23 | -45681.225 | 3729.0543 | 11922.235 | | 9996.455 | | 2.49515 |
| 24 | -43755.445 | 1882.09405 | 1925.78 | | - | | 2.68069 |
| 25 | -46838.17 | 6164.39434 | -4487.155 | | 7231.19 | | - |
| Method | Best *K* |  | |  | |  |  |
| Evanno | 3 |  | |  | |  |  |
| Pritchard | 3 |  | |  | |  |  |
