# Supplementary material for: Low genetic differentiation among morphologically distinct Cycas species informs the delineation of conservation management units
Source: Ann Bot. 2025 Nov 13;137(2):415–30. doi: 10.1093/aob/mcaf276 (PMC12823241; doi:10.1093/aob/mcaf276)
Supplement: mcaf276_Supplementary_Data [file mcaf276_supplementary_data.zip › Supplementary Table 5.docx]

| **Characters** | **Angle of insertion to ML (°)** | **ML width (mm)** | **ML length (mm)** | **Area Stom. Ap. (μm)** | **LLGCell (μm)** | **WLGCell (μm)** | **LUPolar (μm)** |
| --- | --- | --- | --- | --- | --- | --- | --- |
| ***Cycas armstrongii*** | | | | | | | |
| Min. | 104 | 4.27 | 54.2 | 1244 | 28.2 | 8.99 | 7.88 |
| 1st Qu. | 113 | 5.64 | 102 | 1786 | 40.7 | 13.4 | 10.9 |
| Median | 118 | 6.26 | 125 | 1923 | 44.2 | 14.3 | 11.9 |
| Mean | 117 | 6.13 | 118 | 1911 | 43.5 | 14.1 | 12.0 |
| 3rd Qu. | 121 | 6.63 | 133 | 2134 | 47.3 | 15.5 | 13.0 |
| Max. | 130 | 7.36 | 195 | 2300 | 49.9 | 20.2 | 17.4 |
| ***Cycas maconochiei*** | | | | | | | |
| Min. | 101 | 3.46 | 63.8 | 1303 | 30.1 | 13.4 | 6.27 |
| 1st Qu. | 107 | 4.42 | 87.0 | 1552 | 36.7 | 15.0 | 9.70 |
| Median | 110 | 4.76 | 108 | 1692 | 40.0 | 15.6 | 11.7 |
| Mean | 110 | 4.74 | 105 | 1678 | 39.6 | 16.0 | 11.6 |
| 3rd Qu. | 113 | 5.15 | 125 | 1760 | 43.3 | 16.9 | 13.4 |
| Max. | 120 | 5.79 | 146 | 2170 | 46.1 | 19.1 | 17.1 |
| ***Cycas armstrongii x maconochiei*** | | | | | | | |
| Min. | 113 | 4.36 | 79.9 | 1540 | 39.1 | 11.9 | 9.25 |
| 1st Qu. | 113 | 4.52 | 106 | 1701 | 39.8 | 13.3 | 9.44 |
| Median | 118 | 4.82 | 120 | 1770 | 40.6 | 14.9 | 10.9 |
| Mean | 120 | 4.84 | 111 | 1798 | 41.1 | 15.3 | 11.2 |
| 3rd Qu. | 125 | 5.13 | 125 | 1867 | 41.9 | 16.9 | 12.7 |
| Max. | 130 | 5.35 | 125 | 2110 | 44.3 | 19.4 | 13.6 |

**Supplementary Table 5 Summary of morphological character measurements for *C. armstrongii*,** *Cycas* ***maconochiei*** **subsp. *maconochiei*** **and *C. armstrongii* x *maconochiei*.** The results summarise each of the measurements for each micromorphological and macromorphological character analysed for *C. armstrongii* (3 samples per population), *C. maconochiei* subsp. *maconochiei* (3 samples per population) and *C. armstrongii* x *maconochiei* (3 samples total) and include a total of seven characters: Angle of insertion to ML = angle of insertion of the median leaflet to the rachis (°), ML width = width of the median leaflet (mm), ML length = length of the median leaflet (mm), Area Stom Ap = area of the stomatal apparatus (μm), LLGCell = length of left guard cell (μm), WLGCell = width of left guard cell (μm) and LUPolar = length of upper polar extension (μm).
