# Supplementary material for: Low genetic differentiation among morphologically distinct Cycas species informs the delineation of conservation management units
Source: Ann Bot. 2025 Nov 13;137(2):415–30. doi: 10.1093/aob/mcaf276 (PMC12823241; doi:10.1093/aob/mcaf276)
Supplement: mcaf276_Supplementary_Data [file mcaf276_supplementary_data.zip › Supplementary Table 6.docx]

**Supplementary Table 6**. Pairwise T-test showing micromorphological/macromorphological variation of seven characters between *C. armstrongii*, *C. maconochiei* subsp. *maconochiei* and *C. armstrongii* x *maconochiei*: Angle of insertion to ML = angle of insertion of the median leaflet to the rachis, ML width = width of the median leaflet, ML length = length of the median leaflet, Area Stom Ap = area of the stomatal apparatus, LLGCell = length of leaf guard cell, WLGCell = width of left guard cell and LUpolar = length of upper polar extension.

| **Species** | ***C. armstrongii* vs. *C. maconochiei*** | ***C. armstrongii* vs. *C. armstrongii* x *maconochiei*** | ***C. maconochiei* vs. *C. armstrongii* x *maconochiei*** |
| --- | --- | --- | --- |
| **Angle of insertion to ML** | t = 4.4253 | t = -0.59966 | t = -2.3127 |
|  | df = 47.984 | df = 3.6268 | df = 3.3428 |
|  | p-value = 5.53e-05 | p-value = 0.5842 | p-value = 0.09476 |
| **ML width** | t = 7.1975 | t = 4.7912 | t = -0.35621 |
|  | df = 49.485 | df = 6.0035 | df = 4.99 |
|  | p-value = 3.114e-09 | p-value = 0.003023 | p-value = 0.7363 |
| **ML length** | t = 1.8786 | t = 0.56826 | t = -0.54643 |
|  | df = 49.915 | df = 4.5809 | df = 4.2134 |
|  | p-value = 0.06615 | p-value = 0.5966 | p-value = 0.6124 |
| **Area Stom Ap** | t = 3.4294 | t = 0.87191 | t = -0.96681 |
|  | df = 47.521 | df = 4.4328 | df = 3.7288 |
|  | p-value = 0.00126 | p-value = 0.4279 | p-value = 0.3921 |
| **LLGCell** | t = 2.9319 | t = 1.623 | t = -1.0627 |
|  | df = 49.953 | df = 8.7682 | df = 8.0843 |
|  | p-value = 0.005072 | p-value = 0.1399 | p-value = 0.3186 |
| **WLGCell** | t = -3.288 | t = -0.6796 | t = 0.411 |
|  | df = 46.295 | df = 3.4947 | df = 3.2276 |
|  | p-value = 0.001931 | p-value = 0.5391 | p-value = 0.7069 |
| **LUPolar** | t = 0.59585 | t = 0.73005 | t = 0.3449 |
|  | df = 43.788 | df = 3.9825 | df = 4.8839 |
|  | p-value = 0.5543 | p-value = 0.506 | p-value = 0.7445 |
