# Supplementary figures and images for: Low genetic differentiation among morphologically distinct Cycas species informs the delineation of conservation management units
Source: Ann Bot. 2025 Nov 13;137(2):415–30. doi: 10.1093/aob/mcaf276 (PMC12823241; doi:10.1093/aob/mcaf276)

Delta K

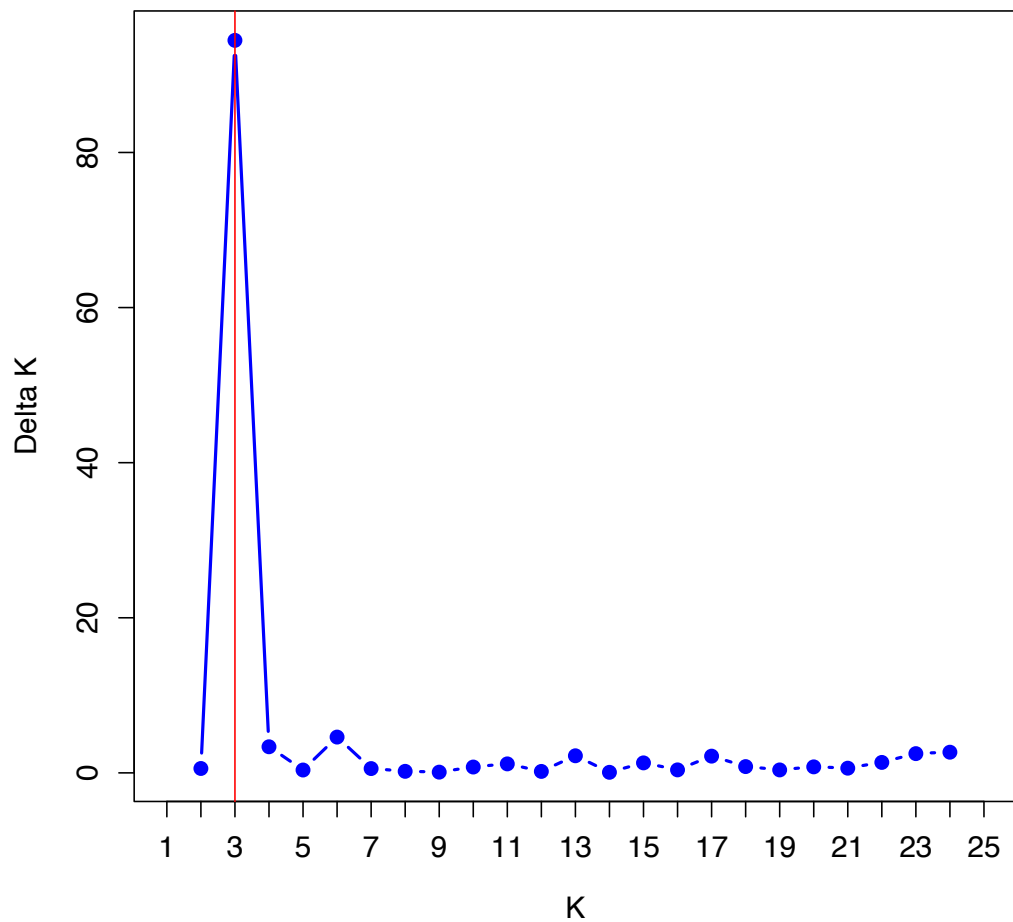

Mean LnP(K)  $\pm$  Stdev

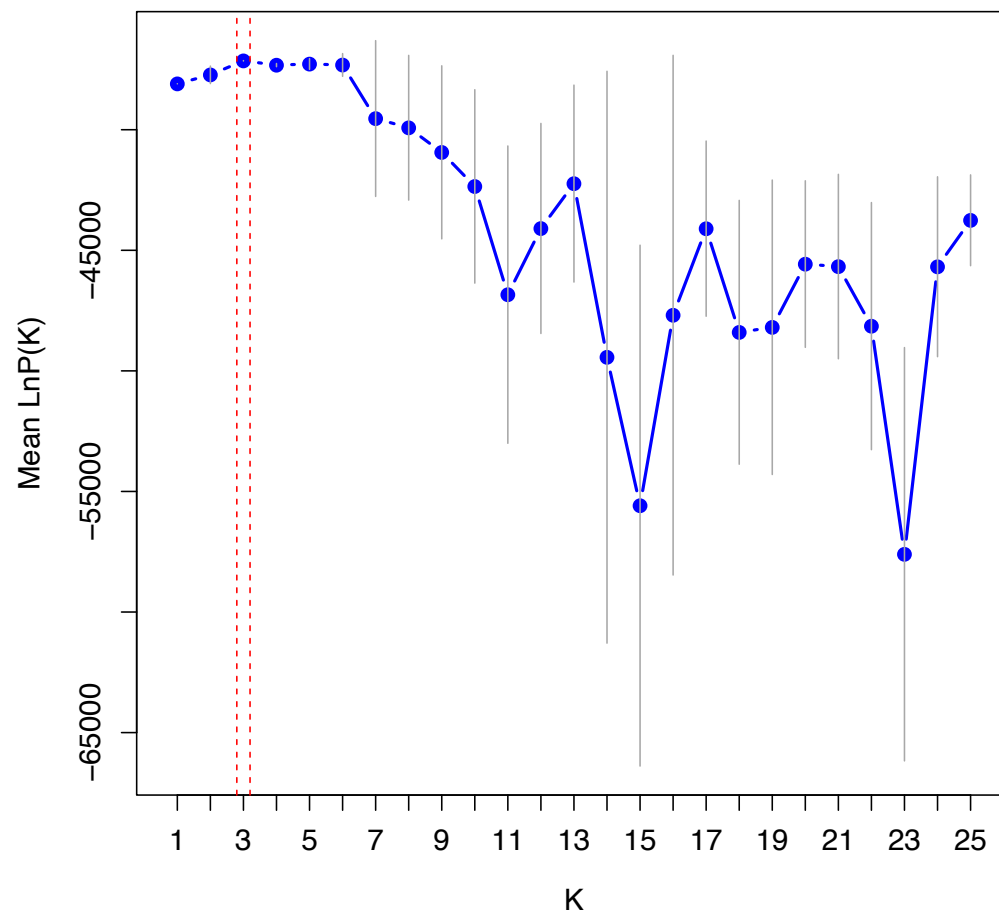

Supplement: mcaf276_Supplementary_Data [file mcaf276_supplementary_data.zip › Supplementary Figure 1.pdf]

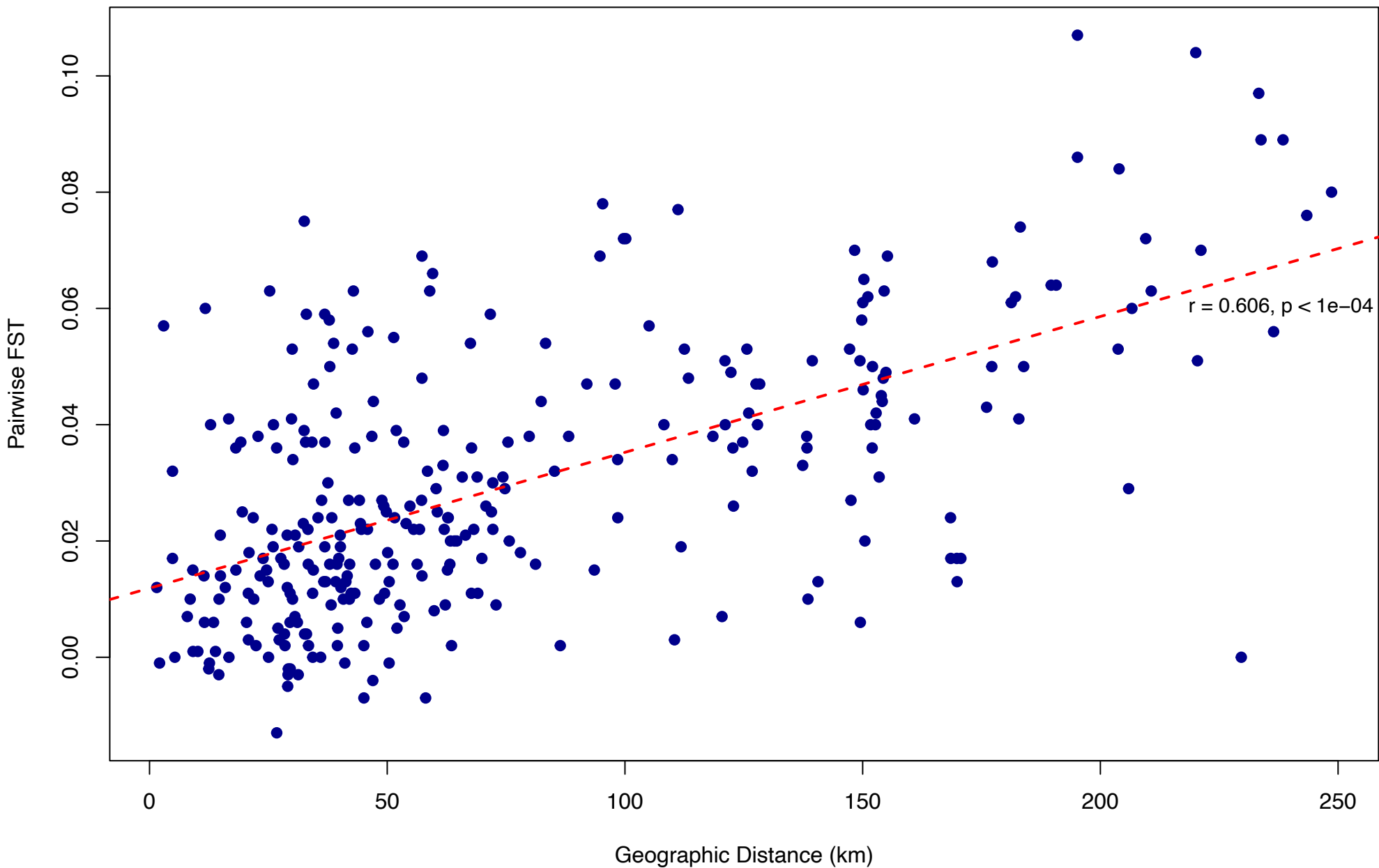

Supplement: mcaf276_Supplementary_Data [file mcaf276_supplementary_data.zip › Supplementary Figure 2.pdf]

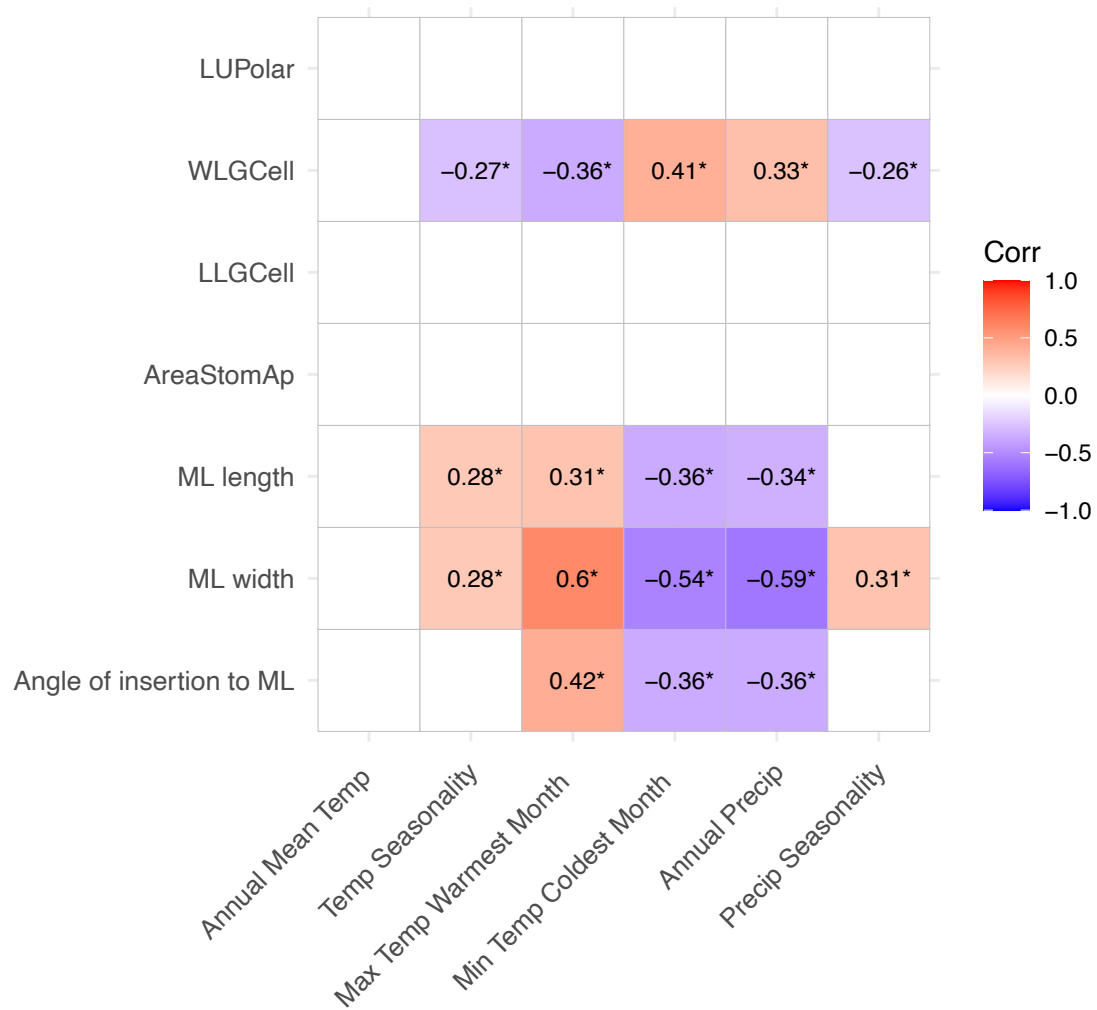

Supplement: mcaf276_Supplementary_Data [file mcaf276_supplementary_data.zip › Supplementary Figure 3.pdf]
